# Herbicide spray drift from ground and aerial applications: Implications for potential pollinator foraging sources

**DOI:** 10.1038/s41598-022-22916-4

**Published:** 2022-10-26

**Authors:** Thomas R. Butts, Bradley K. Fritz, K. Badou-Jeremie Kouame, Jason K. Norsworthy, L. Tom Barber, W. Jeremy Ross, Gus M. Lorenz, Benjamin C. Thrash, Nick R. Bateman, John J. Adamczyk

**Affiliations:** 1 Department of Crop, Soil, and Environmental Sciences, University of Arkansas System Division of Agriculture, 2001 Hwy 70 E, Lonoke, AR 72086 USA; 2 USDA-ARS Aerial Application Technology Research Unit, 3103 F&B Road, College Station, TX 77845 USA; 3 Department of Crop, Soil, and Environmental Sciences, University of Arkansas-Fayetteville, 1366 W Altheimer Dr, Fayetteville, AR 72704 USA; 4 Department of Entomology and Plant Pathology, University of Arkansas System Division of Agriculture, 2001 Hwy 70 E, Lonoke, AR 72086 USA; 5 Department of Entomology and Plant Pathology, University of Arkansas System Division of Agriculture, 2900 Hwy 130 E, Stuttgart, AR 72160 USA; 6 USDA-ARS, 141 Experiment Station Rd, Stoneville, MS 38776 USA

**Keywords:** Flowering, Environmental impact

## Abstract

A field spray drift experiment using florpyrauxifen-benzyl was conducted to measure drift from commercial ground and aerial applications, evaluate soybean [*Glycine max* (L.) Merr.] impacts, and compare to United States Environmental Protection Agency (US EPA) drift models. Collected field data were consistent with US EPA model predictions. Generally, with both systems applying a Coarse spray in a 13-kph average wind speed, the aerial application had a 5.0- to 8.6-fold increase in drift compared to the ground application, and subsequently, a 1.7- to 3.6-fold increase in downwind soybean injury. Soybean reproductive structures were severely reduced following herbicide exposure, potentially negatively impacting pollinator foraging sources. Approximately a 25% reduction of reproductive structures up to 30.5-m downwind and nearly a 100% reduction at 61-m downwind were observed for ground and aerial applications, respectively. Aerial applications would require three to five swath width adjustments upwind to reduce drift potential similar to ground applications.

## Introduction

With 366 million hectares treated globally, synthetic auxin herbicides (WSSA Group 4) are the third most frequently used herbicide site-of-action behind acetolactate synthase-inhibitors (WSSA Group 2) and 5-enolpyruvylshikimate-3-phosphate synthase-inhibitors (WSSA Group 9)^1^. Their extensive use for selective broadleaf weed management started with the introduction of 2,4-D (2,4-dichlorophenoxyacetic acid) in the mid-1940s^1^ and have been frequently used in rice (*Oryza sativa* L.) production systems^2^. Recently, synthetic auxin herbicide use has further increased due to herbicide resistance concerns^3^ and the introduction of soybean [*Glycine max* (L.) Merr] and cotton (*Gossypium hirsutum* L.) cultivars resistant to dicamba^4^ and 2,4-D^5^. Synthetic auxin herbicides are classified as aryloxyacetates (2,4-D, MCPA, dichlorprop, mecoprop, triclopyr, and fluroxypyr), benzoates (dicamba), quinoline-2-carboxylates (quinclorac and quinmerac), pyrimidine-4-carboxylates (aminocyclopyrachlor), pyridine-2-carboxylates (picloram, clopyralid, and aminopyralid), and 6-aryl-picolinate herbicides (Arylex™ active and Rinskor™ active)^6^. Florpyrauxifen-benzyl [benzyl 4-amino-3-chloro-6-(4-chloro-2-fluoro-3-methoxyphenyl)-5-fluoropicolinate] was commercialized in 2018 under the trade name of Loyant™ with Rinskor™ active for weed control in rice^7^. It was initially rapidly adopted by rice growers in the midsouthern US because it can be used to control three of the top five most problematic weeds in rice production, barnyardgrass (*Echinochloa crus-galli* P. Beauv), sedge spp. (*Cyperus* spp.), and Palmer amaranth (*Amaranthus palmeri* S. Wats.)^8,9^.

The use of synthetic auxin herbicides to control problematic weeds has led to numerous herbicide drift injury concerns to neighboring sensitive vegetation and crops^10,11^. In 2017 for example, approximately 1.5 million hectares of dicamba-injured soybeans were reported in the United States^12^. In 2018, off-target movement of florpyrauxifen-benzyl came to the forefront in Arkansas, prompting an advisory statement from the Arkansas State Plant Board^13^. There are multiple avenues for off-target herbicide movement to occur; however, emphasis is typically placed on spray particle drift because management strategies can be implemented to aid in mitigating this form of off-target movement. For example, spray particle drift potential increases with a decrease in droplet size; making droplet size a critical factor for herbicide applications^14^. Application practices and decisions influencing droplet size from aerial and ground spray equipment include nozzle type and size^15^, spray pressure^16^, herbicide formulations^17^, and spray mixtures^18^. In Arkansas, ground application equipment accounts for 49% of herbicide applications on reported agronomic crop hectares while aerial application equipment is used for 51% of herbicide applications^19^. Therefore, understanding the impact of each application method on herbicide spray drift, particularly synthetic auxins such as florpyrauxifen-benzyl, is critical.

Pollinators are imperative for global agricultural production. In the United States, annual pollination services for all crops that require direct pollination account for more than US$15 billion with wild bee communities accounting for approximately US$3.5 billion of these pollination services^20^. Unfortunately, pollinator populations are declining^21^ due to multiple stressors^22^ among which insecticide use and insufficient forage are the two primary stressors for pollinators in agroecosystems^23^. Herbicides have been shown to reduce flower production and delay flowering^24^, as well as reduce nectar sources and floral density up to 85% which might impact pollinator visitation^25^. Soybean flowers can be a source of nectar and pollen for various visiting pollinators^26^. Its pollen was found on up to 38% of bees examined by Gill and O’Neal^27^. However, soybean is sensitive to multiple synthetic auxin herbicides, including sublethal rates of florpyrauxifen-benzyl^28^. For example, a sublethal rate of florpyrauxifen-benzyl applied at R4 and R5 stages induced 15 and 24% yield reduction for the offspring, respectively^29^. However, research investigating the actual spray drift of florpyrauxifen-benzyl from ground and aerial application equipment and the subsequent impact on soybean and potential pollinator foraging sources is lacking. Yet, this information can improve predictions of auxin herbicide drift concerns and help growers reduce off-target movement.

The United States Environmental Protection Agency (US EPA) guidelines for herbicide label generation and assessing potential drift risks include the use of spray droplet size data and computer simulation models, including AgDRIFT and AgDISP. AgDRIFT is a modified version of AgDISP that serves as an initial screening model for estimating downwind deposition from ground, aerial, and orchard/vineyard applications. The Agricultural DISPersal (AgDISP) model allows for more detailed input conditions and higher-level modeling of aerial and ground spray applications^30–32^. The model was previously used by Fritz et al.^17^ to evaluate the effect of changes in droplet size, resulting from variations in airspeed, on downwind movement. The model has undergone continuous improvements, adding notable features that improved the speed and accuracy of predictions^33–35^, and its use has been expanded to model ground-based spray applications drift potential in addition to aerial applications. However, validation of these models with physically collected spray drift data and injury to downwind susceptible plant species is needed.

The first objective of this research was to measure physical spray drift of florpyrauxifen-benzyl and compare the off-target spray movement from commonly used ground and aerial application equipment. The second objective was to evaluate the downwind herbicide spray drift impact on susceptible soybean, specifically growth and reproductive structures, to assess potential influences on pollinator foraging sources. The final objective of this research was to compare measured downwind drift deposits versus predicted downwind drift deposits from AgDISP.

## Results and discussion

### Field spray drift experiment

**Figure 1:**
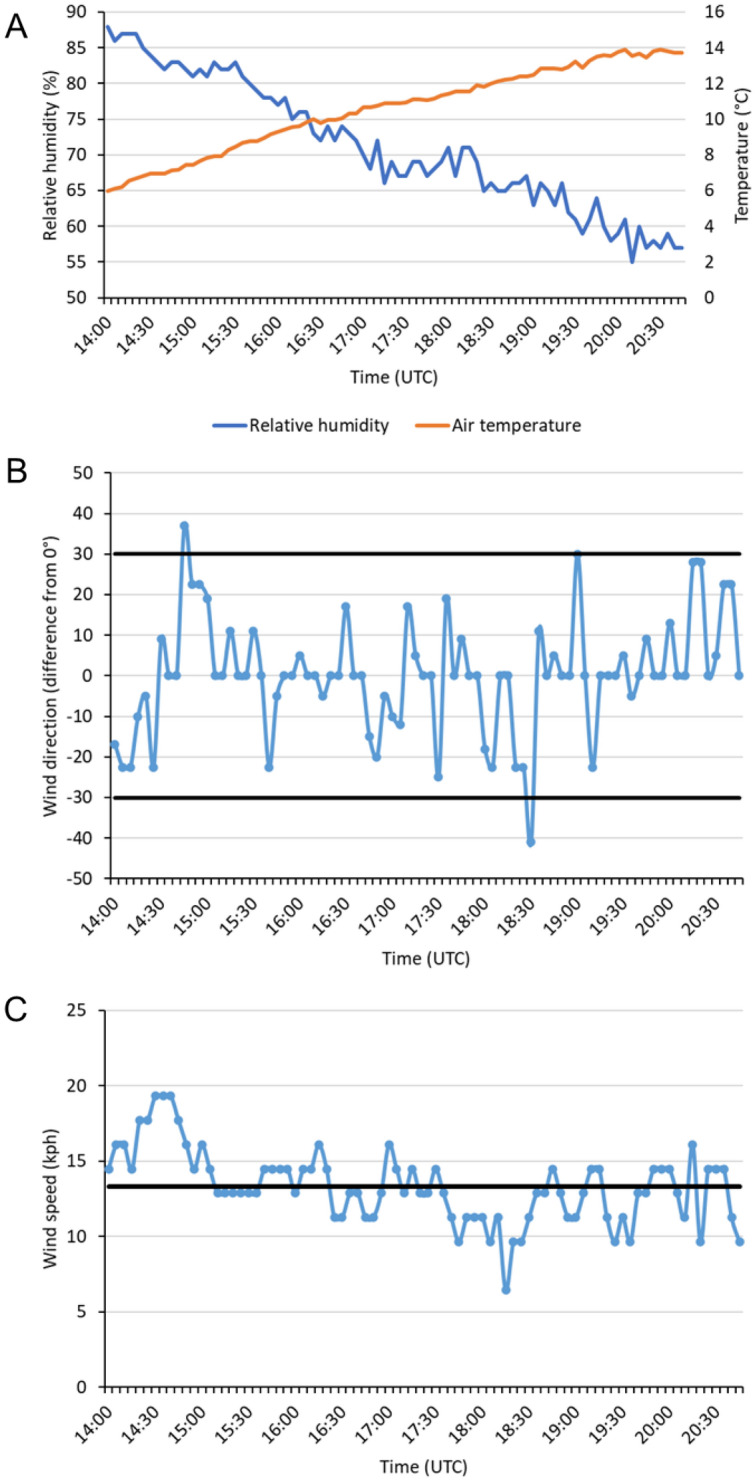
Meteorological data collected throughout the duration of the spray drift field experiment including air temperature and relative humidity (**A**), wind direction deflection from 0°(**B**), and wind speed (**C**). The horizontal black lines in (**B**) represent the 30° maximum wind direction deviation permitted under guidelines established by the US EPA^36^. The horizontal black line in (**C**) represents the average wind speed over the duration of the experiment (13 kph).

The field spray drift experiment was conducted under optimal meteorological conditions in accordance with guidelines established by the US EPA^36^. Throughout the duration of the experiment, air temperature ranged between 6 and 14 °C, relative humidity ranged from 55 to 88%, wind direction deviated less than 30° from the established collection line (excluding two individual points in time), and wind speed averaged 13 kph (Fig. 1).

**Figure 2:**
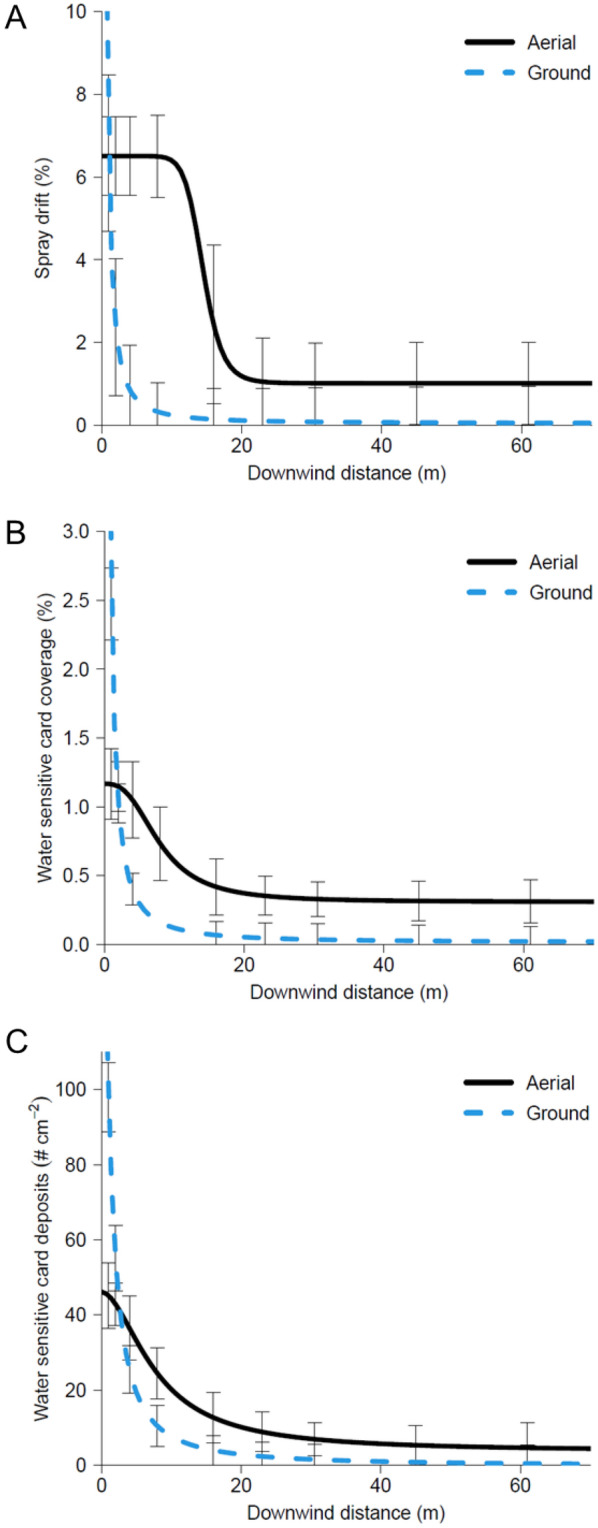
Mylar card spray drift deposition (**A**), water sensitive card coverage (**B**), and water sensitive card number of deposits (**C**) modeled using four parameter log-logistic regressions to evaluate measured spray drift from ground and aerial application equipment.

**Figure 3:**
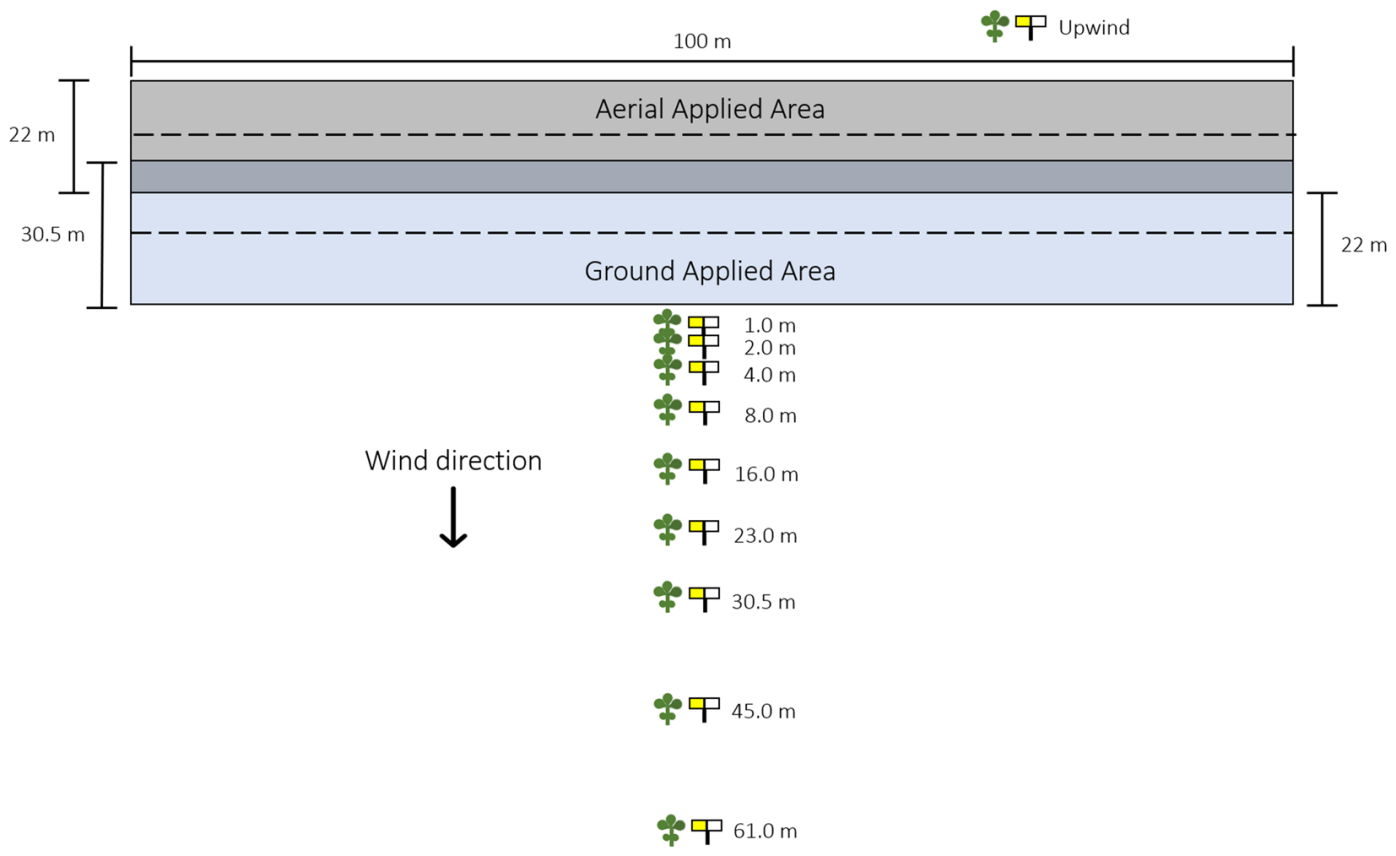
Diagram of the spray drift field experiment setup including the intended applied area of each application method (black = aerial, blue = ground) and the 10 collection station locations (nine downwind, one upwind).

Four-parameter log-logistic regression parameter estimates for each data response variable as a function of downwind distance are presented in Table 1. Spray drift deposition on Mylar cards (expressed as a percent of the total theoretical applied), water sensitive card coverage (%), and water sensitive card deposits (# cm^−2^) demonstrated similar responses in downwind drift measurements (Fig. 2). The ground spray application had a steeper slope for each regression meaning spray drift more rapidly declined as downwind distance increased compared to the aerial spray application (Table 1, Fig. 2). Additionally, even with one full swath width adjustment upwind for the aircraft spray pass (Fig. 3), the aerial application had greater downwind spray drift deposits compared to the ground application, and spray drift never reached zero at the farthest downwind collection station (61-m) (Fig. 2).

**Table 1:** Parameter estimates for four parameter log-logistic regressions used to model spray drift and resulting soybean response from ground and aerial applications of florpyrauxifen-benzyl.

	Application	b (SE)	c (SE)	d (SE)	e (SE)
**Spray drift**					
Mylar card spray drift deposition	Aerial	10.63 (26.95)	1.01 (0.50)	6.51 (0.48)	14.49 (3.55)
	Ground	1.53 (0.63)	0.05 (0.46)	198.67 (-)	0.11 (-)
Water sensitive card coverage	Aerial	2.72 (2.36)	0.31 (0.09)	1.17 (0.14)	7.91 (2.38)
	Ground	1.44 (-)	0.01 (0.05)	15.87 (-)	0.31 (-)
Water sensitive card # of deposits	Aerial	1.82 (1.13)	3.65 (5.34)	46.16 (6.49)	7.82 (2.14)
	Ground	1.40 (0.47)	-0.20 (3.11)	185.07 (106.98)	1.09 (0.88)
**Soybean**^**a**^					
Visual injury	Aerial	2.89 (1.03)	0.00	100.00	86.20 (14.72)
	Ground	1.76 (0.19)	0.00	100.00	30.49 (1.49)
Canopy coverage reduction	Aerial	2.84 (1.15)	0.00	100.00	93.18 (20.95)
	Ground	2.76 (0.48)	0.00	100.00	30.07 (1.74)
Reproductive structures reduction	Aerial	0.37 (0.11)	0.00	100.00	70,935.38 (-)
	Ground	4.73 (1.42)	0.00	100.00	24.02 (1.50)

^a^Parameters “c” and “d” were fixed at 0 and 100, respectively, for soybean measurements as all data were bound between 0 and 100%.

Predicted downwind distances in which Mylar card spray drift deposition, water sensitive card coverage, and water sensitive card deposits were reduced by 25, 50, and 90% (PD_25_, PD_50_, and PD_90_, respectively) were determined from the four-parameter log-logistic regressions and are presented in Table 2. Averaged across the three data collection methods, the aerial application resulted in a PD_25_, PD_50_, and PD_90_ of 7.55-, 10.07-, and 20.54-m, respectively. The ground application resulted in an average PD_25_, PD_50_, and PD_90_ of 0.23-, 0.50-, and 2.36-m, respectively. As a result, the aerial application resulted in an 8.7- to 32.7-fold increase in downwind spray drift compared to the ground application when including one upwind swath width adjustment. However, when comparing the application methods using the best fitting regression where all parameter estimates had calculable standard errors (water sensitive card # of deposits), the aerial application resulted in a 5.0- to 8.6-fold increase in downwind spray drift.

**Table 2:** Predicted downwind distances to observe 25, 50, and 90% reductions in spray drift potential (PD_25_, PD_50_, and PD_90_, respectively) derived from four parameter log-logistic regressions used to model spray drift and resulting soybean response from ground and aerial applications of florpyrauxifen-benzyl.

	Application	PD_25_ (SE)	p-value	PD_50_ (SE)	p-value	PD_90_ (SE)	p-value
**Spray drift**							
Mylar card spray drift deposition	Aerial	13.07 (6.50)	0.004	14.49 (3.55)	< 0.0001	17.82 (5.54)	< 0.0001
	Ground	0.05 (-)		0.11 (-)		0.46 (-)	
Water sensitive card coverage	Aerial	5.29 (3.04)	0.027	7.91 (2.38)	< 0.0001	17.73 (10.44)	< 0.0001
	Ground	0.14 (-)		0.31 (-)		1.42 (-)	
Water sensitive card # of deposits	Aerial	4.28 (2.18)	< 0.0001	7.82 (2.14)	< 0.0001	26.06 (18.99)	< 0.0001
	Ground	0.50 (0.53)		1.09 (0.88)		5.21 (1.88)	
**Soybean**							
Visual injury	Aerial	58.97 (3.95)	< 0.0001	86.20 (14.72)	< 0.0001	184.18 (79.74)	0.1066
	Ground	16.36 (1.33)		30.49 (1.49)		105.92 (14.99)	
Canopy coverage reduction	Aerial	63.27 (7.13)	< 0.0001	93.18 (20.95)	< 0.0001	202.10 (105.70)	0.0003
	Ground	20.19 (1.72)		30.07 (1.74)		66.71 (10.36)	
Reproductive structures reduction	Aerial	3727.46 (-)	< 0.0001	70,935.38 (-)	< 0.0001	25,690,000.00 (-)	< 0.0001
	Ground	19.04 (2.06)		24.02 (1.50)		38.24 (5.11)	

Previous aerial application spray drift research has indicated a range in downwind spray drift deposits from 0.5% of the field-applied rate 150-m downwind^37^to 1% of the field-applied rate deposited up to 500-m downwind of the field boundary^38^. Results amongst aerial spray drift research trials likely vary due to the droplet size emitted from the aircraft, as well as other external meteorological factors (wind speed, temperature, and humidity) that have been previously noted to affect off-target spray movement and enhance variability amongst aerial spray drift trials^38,39^. The major factors that impact both aerial and ground spray drift include wind speed, release height, and droplet size^40–43^. Considering the wind speed and droplet size were nearly identical within this research, the increase in observed downwind spray deposition from the aerial application compared to the ground application may have resulted from the increase in release height and the greater percentage of spray volume contained in fine droplets (Table 3). This was previously demonstrated as other research determined that doubling the boom height from a ground application resulted in a three-fold increase in downwind spray drift^44^. In the present research, the aerial application flight height was approximately five times higher than the ground application boom height, which would equate to a theoretical 7.5-fold increase in spray drift. This theoretical spray drift potential increase falls within the observed 5.0- to 8.6-fold increase in downwind spray drift from the present experimental research. Additional factors such as the wind profile with regard to release height, spray pattern formation, and airflow vortex effects may have influenced the off-target spray movement of the aerial application compared to the ground application. Future research should directly investigate the impact of these factors on drift from aerial applications and determine an optimum flight height for herbicide applications.

**Table 3:** Spray droplet size summary data from ground and aerial nozzles used in field spray drift experiment.

Nozzle	Orifice	D_v10_ (μm)	D_v50_ (μm)	D_v90_ (μm)	% Volume < 100 μm	Droplet Size Classification^a^
CP 09 Straight Stream	0.078	131	355	666	6.2	Medium
CP 09 Straight Stream	0.125	161	374	647	3.5	Coarse
CP 09 Composite Volume Weighted	n/a	148	366	656	4.7	Coarse
ER110	10	189	369	589	1.4	Coarse

^a^As established by *Spray Nozzle Classification by Droplet Spectra*, ASABE S572.3 (St. Joseph, MI: American Society of Agricultural and Biological Engineers, 2020) and data from laboratory wind tunnel generated reference nozzle data.

Aerial applicators would benefit from understanding these spray drift dynamics and utilizing tools to determine an optimal upwind swath width adjustment to reduce off-target spray impacts^43^. In this research with a synthetic auxin herbicide (florpyrauxifen-benzyl), approximately three to five full swath width adjustments upwind (rather than only one like that used in the present research) would be required to reduce spray drift potential similar to the ground application. Results from the modelling efforts in the present research that examined multiple, consecutive spray passes confirmed this determination and is further discussed later in this paper. Additionally, ground applicators could use these results to implement further drift mitigation strategies such as increasing droplet size^40^ and/or integrating upwind spray adjustments or barriers to increase the downwind distance to susceptible crops^42^.

### Soybean

Soybean plants were extremely sensitive to florpyrauxifen-benzyl (Loyant®, Corteva Agriscience, Indianapolis, IN USA), with injury visible within three days following exposure (personal observations). Soybean injury can be directly correlated to spray droplet drift deposits because florpyrauxifen-benzyl has a low vapor pressure (3.2 × 10^−5^ Pa at 20 °C and 4.6 × 10^−5^ Pa at 25 °C) and as such, has been deemed not volatile^45^. Visual estimations of injury (%), plant canopy coverage (% reduction from nontreated control), and number of reproductive structures (% reduction from nontreated control) were collected 35 days after exposure (DAE). Four-parameter log-logistic regression parameter estimates for each soybean data response variable are presented in Table 1.

**Figure 4:**
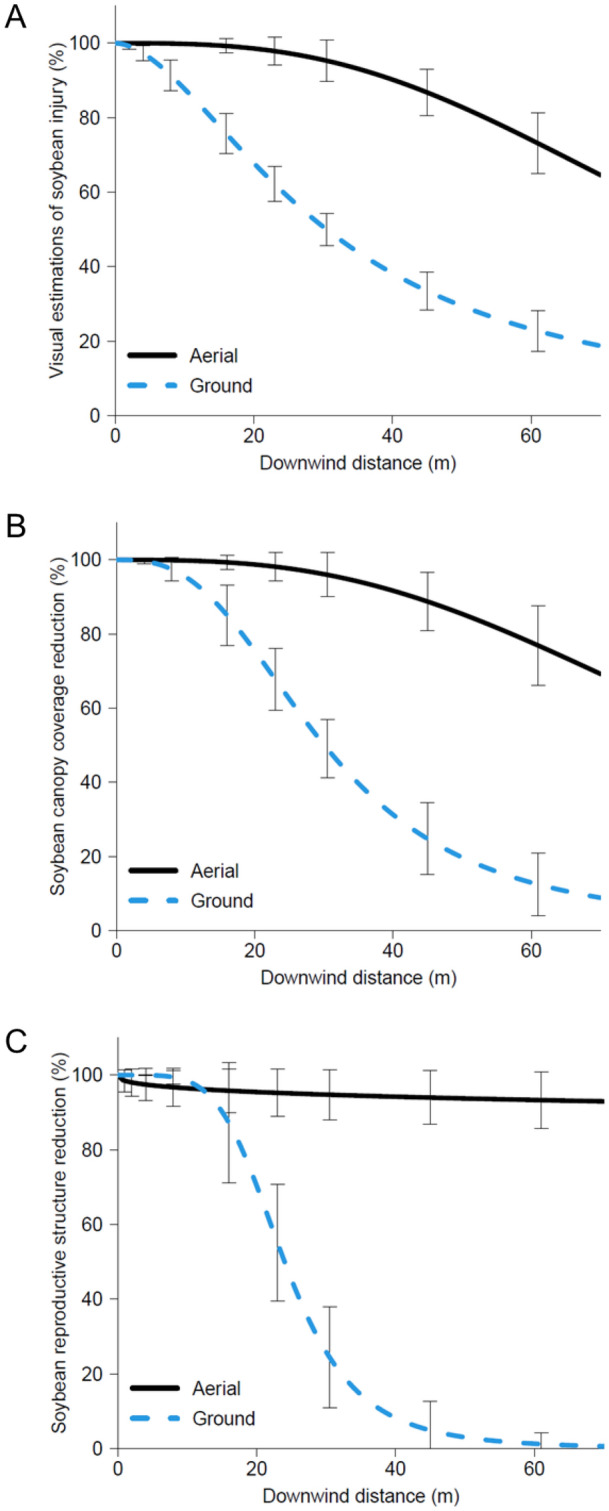
Visual estimations of soybean injury (**A**), soybean canopy coverage reduction (**B**), and soybean reproductive structure (flowers and pods) reduction (**C**) 35 days after exposure modeled using four parameter log-logistic regressions to evaluate measured spray drift from ground and aerial application equipment. The nontreated control plants averaged 54% canopy coverage and 101 total number of reproductive structures per plant.

Visual estimations of injury showed the ground application resulted in reduced soybean injury at shorter distances downwind compared to the aerial application (Fig. 4). The aerial application resulted in greater than 70% visual soybean injury at the farthest downwind collection station (61 m), while the ground application resulted in approximately 25% injury at the same collection station. The PD_25_, PD_50_, and PD_90_ for visual estimations of soybean injury, derived from the four-parameter log-logistic regressions as a function of downwind distance, were 3.6-, 2.8-, and 1.7-fold larger for the aerial application compared to the ground application (Table 2). The predicted downwind distances in which visual injury would be reduced by 90% were 184- and 106 m for the aerial and ground applications, respectively, both of which were beyond the final collection station used in this research.

Canopy coverage analysis using digital imagery of the soybean plants prior to florpyrauxifen-benzyl exposure resulted in no differences across application type (*p* = 0.9475) with an average of 7.1% canopy coverage indicating no pre-populated bias in soybean growth across treatments (data not shown). At 35 DAE, the nontreated control plants averaged 54% canopy coverage per plant (data not shown). The canopy coverage analysis produced comparable results to the visual estimations of injury response variable (Fig. 4). At 61 m downwind, the aerial application resulted in greater than 75% canopy coverage reduction, while the ground application resulted in approximately 15% canopy coverage reduction at the same collection station (Fig. 4). The PD_25_, PD_50_, and PD_90_ for canopy coverage reduction were 3.1-, 3.1-, and 3.0-fold larger for the aerial application compared to the ground application (Table 2). The predicted downwind distances to achieve only a 10% reduction in canopy coverage (PD_90_) as a result of florpyrauxifen-benzyl exposure were 202- and 67 m for the aerial and ground applications, respectively, both of which were beyond the final collection station used in this research.

Soybean reproductive structures (flowers and pods) were severely impacted 35 DAE of florpyrauxifen-benzyl from both ground and aerial applications (Fig. 4). The nontreated control plants averaged 101 total number of reproductive structures (flowers and pods) per plant 35 DAE (data not shown). The percent reduction of soybean reproductive structures had a steeper decreasing slope compared to the other soybean measurements for the ground application (Table 1, Fig. 4) indicating the occurrence of visual injury and canopy coverage reduction did not necessarily result in a reduction of reproductive structures. However, visual injury and canopy coverage reduction were required to be approximately 30% or less (45 m downwind) for no loss in reproductive structures to occur.

The aerial application had minimal change in the reduction of soybean reproductive structures at further downwind distances; at 61 m downwind, there was still nearly 100% reduction of reproductive structures (Fig. 4). Due to the minimal change in soybean reproductive structures across downwind distances for the aerial application, the four-parameter log-logistic regression was a poor fitting model and resulting downwind distance predictions were nonsensical (Tables 1 and 2). In contrast, the ground application model fit extremely well to the soybean reproductive structure reduction data (Table 1). The PD_25_, PD_50_, and PD_90_ values for soybean reproductive structures were 19.04-, 24.02-, and 38.24 m, respectively (Table 2). These values highlight the potential severe negative impact on pollinator foraging sources as even up to approximately 40 m downwind, reproductive structures (flowers) can be reduced by 10% (PD_90_) following exposure to a synthetic auxin herbicide like florpyrauxifen-benzyl.

The severe soybean injury across response variables was observed further downwind than measured spray drift deposits. The fluorometry analysis had a detection limit of 0.015 ppm of 3, 6, 8-pyrene tetra sulfonic acid tetra sodium salt (PTSA) tracer dye equivalent to a 0.0002 g ai ha^-1^ rate of florpyrauxifen-benzyl (data not shown). Therefore, the difference between deposition detection and soybean injury may be attributed to two things: (1) injury to soybean is possible following exposure to exceptionally low doses of synthetic auxin herbicides, in this instance florpyrauxifen-benzyl, and/or (2) the fine spray drift droplets may have deposited more efficiently on the vertical structures of the soybean plant compared to the horizontal lying Mylar and water sensitive cards^46^.

Previous research has shown the potential injury capable for soybean and other broadleaf crops exposed to reduced rates of florpyrauxifen-benzyl^28,29,47^. The injury observed in the present research was more severe at lower rates compared to the previous reports, which may be due to the dynamics of actual spray drift compared to spraying herbicides at reduced rates over-the-top of plants to simulate drift. Downwind herbicide spray drift would be composed of much finer, more concentrated droplets than what would be present in a direct spray of reduced rates. The finer and more concentrated droplets would more likely be captured and adhere to the vertical plant surfaces resulting in increased injury^46,48^.

The soybean injury observed in this research corroborates previous observations in which delays in peak flowering and reduction in overall flower production from wild plant species occurred following exposure to several herbicides^10^. Additionally, in a constructed native vegetative habitat, research showed that another synthetic auxin herbicide, dicamba, reduced the number of seed heads and pods per plant for several plant species^11^. For *Prunella vulgaris* L. subsp. *lanceolata* (W. Bartram), total inflorescence number was unaffected following exposure to dicamba; however, approximately 15 to 45% of those inflorescences were considered atypical^11^.

The negative floral impact of herbicide off-target movement is also critical for pollinator foraging. More than 30 different bee species have been identified as visiting and collecting pollen from soybean fields during the growing season^27,49^. Previous research demonstrated plants were visited less frequently by pollinators following exposure to sublethal (simulated drift) dicamba rates^25^. As a result, reduced soybean yields may result as soybean fields in which pollinators had visited observed increased yields compared to those without pollinators^50^. This may deteriorate more natural plant communities in an effort to make up for the loss in production. Further pollinator research and implementation into current agricultural production practices is needed to enhance biodiversity while maintaining production on a reduced required land area^51^. All of these results combined indicate herbicide spray drift, as observed in this research from both ground and aerial applications, would likely have a negative impact on diverse plant communities and impose a negative impact on pollinator foraging habits.

It should also be noted that injury observed in this research was due a single spray pass; in a real-world application and spray drift scenario, multiple application passes and the exposure of some plants to repeated or chronic spray drift would likely result in even greater injury and reduction of reproductive structures. Future research should investigate and quantify this repeated exposure potential, as well as identify the influence of additional herbicide active ingredients and alternative plant species to develop a database of plant injury and resulting potential impacts on pollinators’ foraging sources.

### Collected drift deposition vs. modeled drift deposition from AgDrift and AgDISP

The droplet size results were as expected, with both the aerial and ground application setups producing Coarse sprays, as specified by the label (Table 3). The use of two orifice sizes in the aerial spray treatments resulted in the CP 09 straight stream 0.078 orifice producing a Medium spray in wind tunnel testing while the 0.128 orifice produced a Coarse spray (Table 3). However, the combined spray cloud delivered from the aircraft was Coarse, as determined by weighting the average of the wind tunnel results for each orifice by their respective total flowrates across the boom (Table 3). The ground application D_v10_ was 189 μm, higher than the 148 μm from the aerial application; however, the D_v50_ values for both application methods were similar at 369 and 366 μm, respectively. The ground sprayer setup generated almost a quarter of the percent fines compared to the aerial sprayer setup (1.4 versus 4.7%), which is reflected in the field collected drift data (Fig. 2).

**Figure 5:**
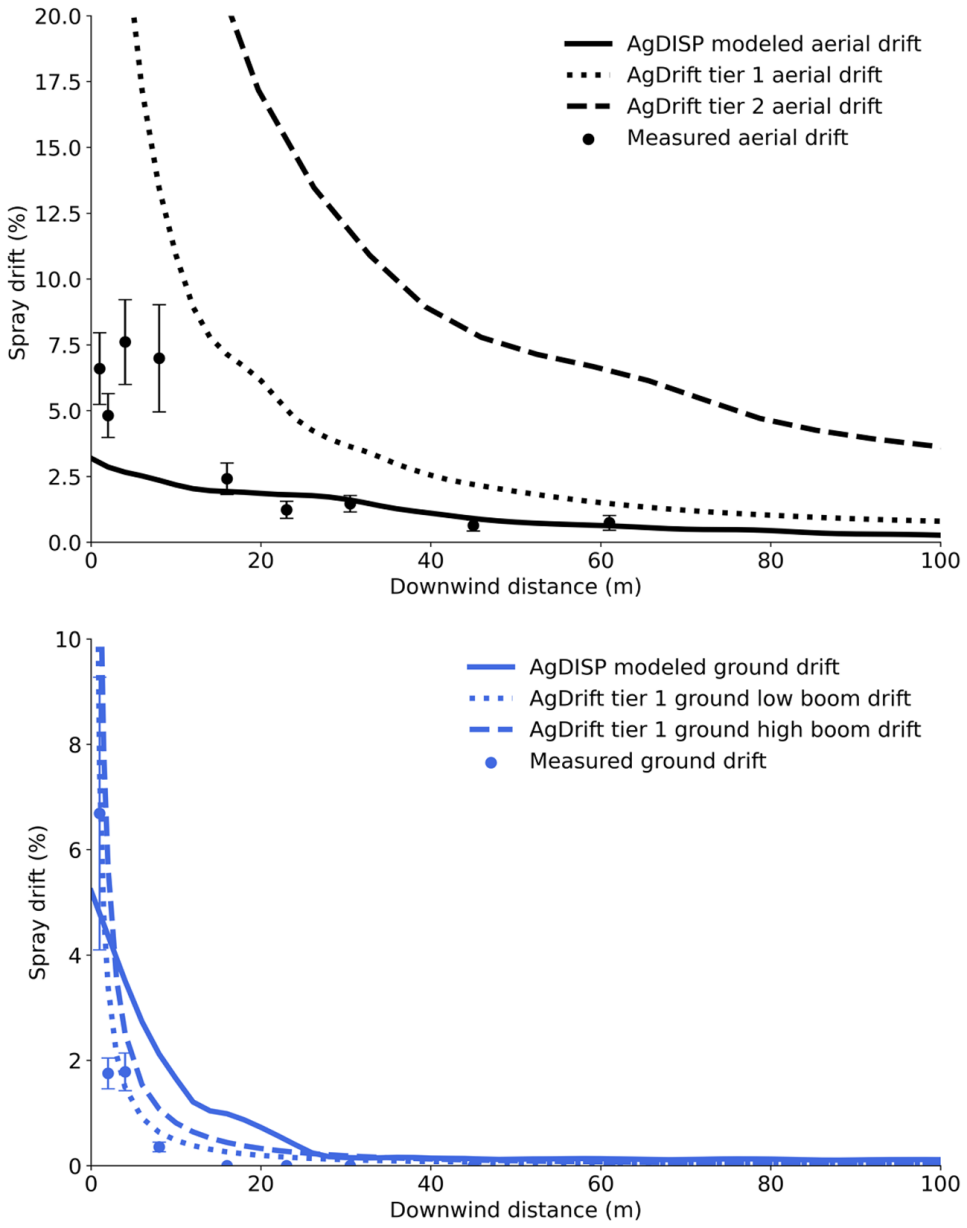
Comparisons of AgDRIFT and AgDISP modeling results to measured data from ground and aerial field trials. Field measured data are presented as the mean percent of application rate at each sampling location with vertical bars as the standard errors.

The AgDRIFT and AgDISP results for the ground boom spray applications corresponded closely to the field measured data, with AgDRIFT’s tier one, low boom results being the closest, followed by the high boom and the AgDISP results. AgDISP overpredicted near-field (< 20 m) deposition (Fig. 5), as previously reported^52,53^. The two models differing is not surprising given that the AgDRIFT results are essentially curve fits to existing field data resulting from trials covering two boom heights and two droplet sized sprays^33^, whereas AgDISP is built on a mechanistic approach to capturing the actual physics involved^52^. These previous efforts also reported that AgDISP underpredicts further downwind; however, the ground field data results generally showed zero deposition due to the low-end sensitivity of the tracer method used.

The differences between the AgDRIFT and AgDISP aerial deposition results were more disparate but expected. The tiered approach implemented within AgDRIFT is intended to provide higher safety margins at the lower tiers through simplified inputs that allow for efficient risk analysis with higher tier levels being used as required^33^. AgDISP has evolved into a model that allows for complete accountability of applied sprays by incorporating established and validated models of aircraft vortices flow field, meteorological transport, canopy interactions, and the physical properties of the spray material^54^. The predicted deposition profiles reflect these considerations with AgDRIFT tier one results greatly over-predicting, followed by the tier two results, and finally the AgDISP predicted deposition data underpredicting in the near field and matching the measured data reasonably well in the far-field (Fig. 5), which was also observed by Bird et al.^55^.

**Figure 6:**
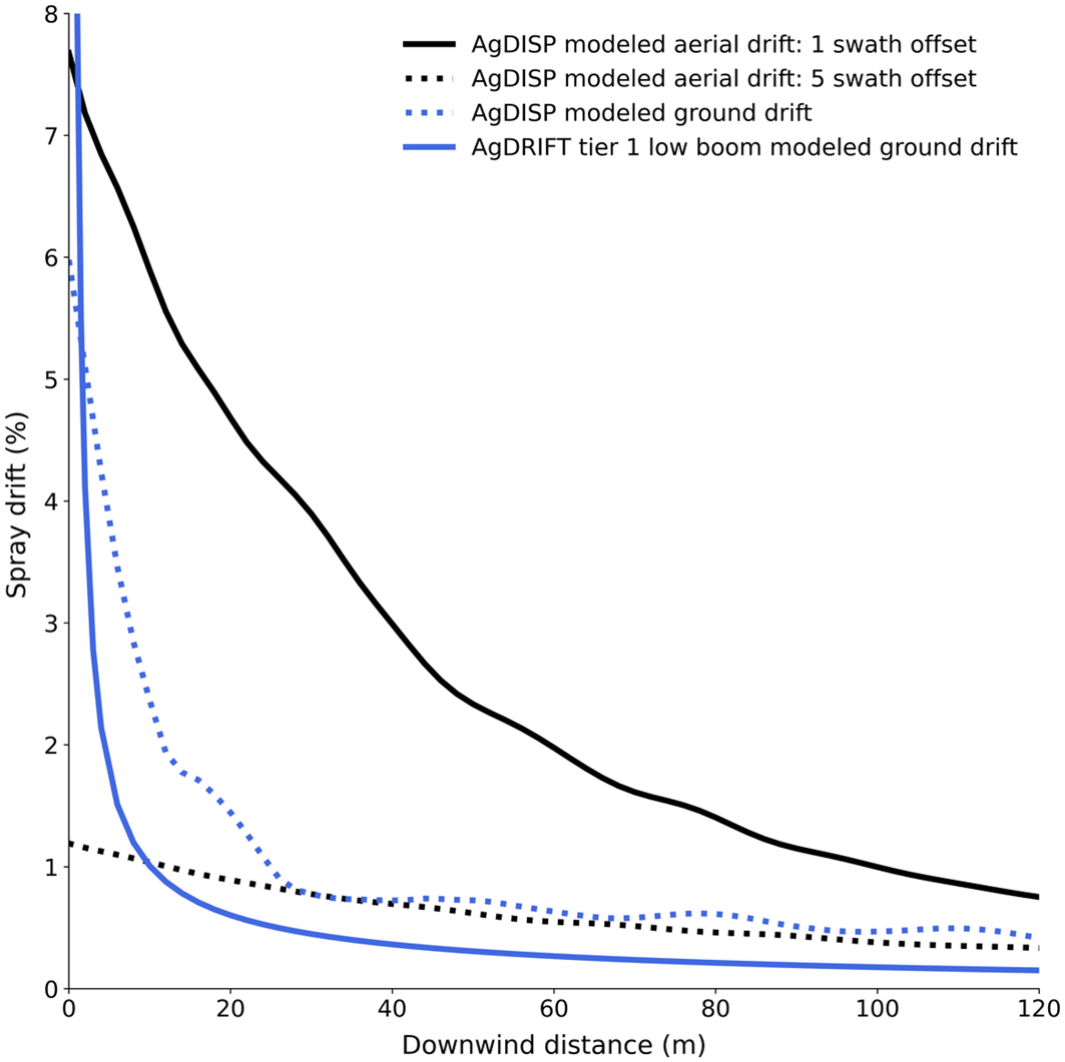
AgDRIFT and AgDISP modeling results for ground and aerial applications under field trial conditions using 20 consecutive passes.

As noted earlier, the field study included only a single spray pass where an actual production application would consist of multiple consecutive passes across a given field. These additional upwind passes would contribute to the cumulative downwind deposits and likely increase both the severity of plant injury and downwind distance at which it occurs. Using the AgDRIFT and AgDISP models, the potential spray drift resulting from 20 consecutive passes was modeled using the same input parameters from the single pass results presented. The addition of additional upwind passes resulted in two to three times the downwind drift compared to the single pass (Figs. 5 and 6). This coupled with the soybean injury results (Fig. 4) would suggest that multiple pass applications of florpyrauxifen-benzyl from both ground and aerial systems, under the same conditions, would result in damage to soybean plants beyond the 60-m sampling position in this study. As previously mentioned, and as supported by modeling, offsetting the aerial application three to five swath widths upwind of the field edge would result in downwind spray drift levels like those from the ground application (Fig. 6).

## Methods

### Field spray drift experiment

A field spray drift experiment was conducted on October 30, 2020, at the Stuttgart Municipal Airport located outside of Stuttgart, AR (34.5974, -91.5751). The experiment consisted of two treatments, ground spray application versus aerial spray application, conducted in a randomized complete block design. The sprayer equipment and application parameters are presented in Table 4. Application parameters were selected based on common practices used by commercial applicators^19^ and by requirements of the florpyrauxifen-benzyl (Loyant®, Corteva Agriscience, Indianapolis, IN USA) herbicide label^8^. The spray solution for both treatments was a mixture of 29.4 g ai ha^−1^ florpyrauxifen-benzyl plus 0.6 L ha^−1^ methylated seed oil (Upland™ MSO, CHS Inc., Inver Grove Heights, MN USA) plus 2.0 g L^−1^ 1, 3, 6, 8-pyrene tetra sulfonic acid tetra sodium salt (PTSA, Spectra Colors Corporation, Kearny, NJ USA) as a tracer dye^56^. The field spray drift experiment was conducted following guidelines established by the US EPA Generic Verification Protocol for Testing Pesticide Application Spray Drift Reduction Technologies for Row and Field Crops^36^ and previous spray drift field experiments^42^. A total of ten replications were conducted. This replicate total was selected for two reasons: (1) it was the maximum number of spray passes that could be completed while meteorological conditions remained consistent, and (2) a power analysis of previous spray drift experiment datasets revealed that a range of 3 to 32 replicates were required for 80% power; as a result, with 8–12 replications, the minimum potential difference in downwind deposition given normal data variability was 10% (B.K. Fritz, unpublished data). Meteorological data were collected on a five-minute interval throughout the duration of the experiment using a Davis Vantage Pro2™ Premium Weather Station (Davis Instruments Corporation, Hayward, CA 94,545 USA).

**Table 4:** Application parameters from the ground and aerial application equipment used in the field spray drift experiment.

	Ground	Aerial
Equipment^a^	Case IH 5550 AimPoint	Air Tractor 802A
Boom/swath width (m)	30.6	21.9
Boom/flight height (m)	0.9	4.6
Pressure (kPa)	276	345
Nozzles^b^	ER11010	CP09 Straight Stream, alternating orifice size pattern (0.78–0.78–0.125, repeating)
Speed (kph)	32	233
Spray volume (L ha^−1^)	94^c^	70

^a^Sprayer manufacturer information: Case IH 5550 AimPoint, CNH Industrial America, LLC. Burr Ridge, IL 60,527 USA; Air Tractor 802A, Air Tractor Inc., Olney, TX 76,374 USA.

^b^Nozzle manufacturer information: ER11010, Wilger Inc., Lexington, TN 38,351 USA; CP09 Straight Stream, Transland, LLC. Wichita Falls, TX 76,302 USA.

^c^The ground spray equipment was a pulse-width modulation sprayer. To achieve 94 L ha^−1^ from the ground spray equipment at 276 kPa, the equipment was pulsed at ~ 70% duty cycle.

Nine downwind (1-, 2-, 4-, 8-, 16-, 23-, 30.5-, 45-, and 61-m) and one upwind (nontreated control) collection stations were established for data collection (Fig. 3). The collection stations were placed on the downwind side from the edge of the ground application spray boom. A 22-m upwind swath adjustment (one full swath width) was used for the aerial application (Fig. 3) as the florpyrauxifen-benzyl label indicates an aerial applicator must compensate for swath displacement in a crosswind environment^8^. Each station comprised three data collection methods: (1) a Mylar card (100 cm^2^) (Grafix Plastics, Cleveland, OH USA) for deposition measurements, (2) a water sensitive card (40 cm^2^) (TeeJet Technologies, Spraying Systems Co., Wheaton, IL USA) for number of deposits and coverage measurements, and (3) a soybean plant (V3-V4 growth stage) as a bioassay measurement. Following each spray pass, a three-minute waiting period was observed to ensure spray droplets had deposited. Mylar cards and water sensitive cards were then immediately collected and placed into pre-labeled plastic zip-top bags. Mylar cards were placed in a dark container to avoid photodegradation of the tracer dye, and water sensitive cards were placed in a cooler to avoid excess humidity from destroying samples. Soybean plants were transported to an area upwind and outside of the experiment area for a minimum of 2 h following application (rainfast period of florpyrauxifen-benzyl)^8^.

Mylar cards were processed using methods established in previous research^42,57^. In brief, spray deposition was determined through fluorometric analysis. Mylar cards were washed using 40 mL of a 9:1 distilled water to isopropyl alcohol (91%) solution. Subsequently, a 1.5 mL aliquot was transferred to a glass cuvette and analyzed using a spectrometer (Flame-S, Ocean Optics, Inc., Largo, FL USA) to detect the PTSA dye fluorescence. Relative fluorescence unit data were then converted to grams of PTSA cm^−2^ (Mylar card) and subsequently percent of theoretical maximum (tank sample concentration) using a calibration curve for the tracer.

Water sensitive cards were digitally scanned (Brother MFC L8900cdw, Brother International Corporation, Bridgewater, NJ USA) with a 1,200 × 2,400 dpi resolution and processed using DepositScan from the United States Department of Agriculture, Agricultural Research Service^58^. Percent coverage and number of deposits cm^−2^ response variables were extracted.

### Greenhouse soybean

Soybean plants used as bioindicators were grown in a greenhouse located at the Lonoke Extension Center in Lonoke, AR. The greenhouse was maintained at a 27/21C day/night temperature, and supplemental light maintained an 11 h daylength for the duration of the experiment. Soybean plants were seeded individually in 2.8-L pots filled with Pro-Mix LP15 potting soil (Premier Tech Ltd., Rivière-du-Loup, Quebec, CA). At planting, each pot was fertilized with Sta-Green All Purpose Plant Food (19.0-6.0-12.0-4.6) (Gro Tec, Inc., Madison, GA USA), and pots were overhead irrigated daily.

Soybean plants were grown to the V3-V4 growth stage before the drift experiment was initiated. Once soybean reached this growth stage, plants were randomly sorted and assigned an application type (ground or aerial), downwind distance, and replication. All soybean plants (including upwind nontreated controls) were transported by covered trailer to the experimental site and exposed to the same environmental conditions. Following completion of the spray drift experiment and a 2 h waiting period was observed, soybean plants were transported back to the greenhouse for the remainder of the evaluation period (35 DAE).

Visual estimations of soybean injury were recorded weekly on a scale of 0 to 100% where 0 indicated no observed visual symptoms and 100 was complete plant death. At 35 DAE, soybean reproductive structures (flowers and pods) were counted and normalized compared to the nontreated control plants for an assessment of percent reduction of total reproductive structure development. Finally, digital images were taken the day prior to the field spray drift experiment and 35 DAE using a 12-MP cellular phone camera (Samsung Galaxy S20 + , Samsung, San Jose, CA USA) affixed at a set height with a tripod. These images were processed and analyzed using FieldAnalyzer (https://turfanalyzer.com/) to detect green pixels providing an estimate of soybean growth and percent canopy coverage^59,60^.

### Droplet size testing

Droplet size testing was conducted in the USDA-ARS Aerial Application Technology Research Unit’s low- and high-speed atomization testing facilities in College Station, Texas. While these facilities and the standard methods used for each were previously documented in detail^61^, a summary is provided here. The nozzles, spray pressures, and spray solutions used in the field studies were evaluated for droplet size using laser diffraction (Sympatec HELOS Vario KR laser diffraction particle size analyzer, Sympatec GmbH, Pulverhaus, Germany; dynamic droplet size range of 18–3500 μm in 31 bins). The ground application nozzle was tested in a low-speed wind tunnel with the nozzle fan sheet oriented perpendicular to the tunnel floor and exiting the nozzle concurrent with tunnel airflow which was set at 6.7 m s^−1^. The airflow is used to minimize the spatial bias that is inherent with using laser diffraction systems^62^. The aerial application nozzle was similarly tested in a high-speed wind tunnel with the airspeed set to the 233 kph used in the field study. The high-speed airflow past the nozzle and exiting spray geometry results in secondary breakup and is the primary factor influencing the resulting droplet size^63^. Each ground and aerial nozzle and pressure combination had a minimum of three replicate measurements. The cumulative volume weighted droplet diameter distributions were exported for use in spray drift modeling efforts using AgDISP, and summary results in the form of D_v10_, D_v50_, and D_v90_ (droplet diameters for which 10, 50, and 90% of the total spray volume is comprised of smaller droplets) along with the fines as percentage total spray volume in droplets less than 100 μm in diameter. Additionally, the droplet size classification for each combination was determined^64^.

### Collected drift deposition vs. modeled drift deposition from AgDRIFT and AgDISP

Both AgDRIFT and AgDISP provide an interface that allows users to specify specific application conditions from which spray transport and fate is modeled. Default input parameters were used, unless otherwise stated below. All modeling was done using AgDRIFT version 2.1.1 and AgDISP version 8.29.

Ground modeling in AgDRIFT requires selecting the Tier 1 Ground interface and either low or high boom height and Very Fine to Fine or Fine to Medium/Coarse droplet size. As the boom height in this work falls between the low and high boom conditions of AgDRIFT (0.51 and 1.27 m, respectively), both settings were modeled^34^. Additionally, the number of swaths was set to one in the extended settings to better match field study conditions. AgDISP requires selecting ground as the application method and specifying nozzle type, which for this study was a flat fan. Spray pressure, release height, number and spacing of nozzles, and total swath width were set to those used in the field study (Table 4). Droplet size data was directly imported as the incremental volume diameter distribution from wind tunnel testing. The meteorology parameters were set to the mean values across the field study treatments, specifically wind speed at 13 kph, wind direction as perpendicular to the spray line, temperature at 10 °C, and relative humidity at 71%. Application rate was set as 94 L ha^−1^ and the spray material set to evaporate. Atmospheric stability was set to moderate and canopy was set as none with a surface roughness of 0.04 m. AgDISP sets a default half swath offset, representing that the spray line is offset upwind of the zero downwind edge by one half the width of the swath input. This default swath offset was changed to zero to reflect field study conditions.

Aerial modeling followed the same process as that for ground, with a few key differences. AgDRIFT provides three tiers for aerial applications. Only tiers one and two were used in the AgDRIFT modeling. Tier three is intended to operate as a full version of AgDISP; however, AgDRIFT has not been updated in recent years while AgDISP has had recent and continual updates. Tier one only allows for choosing one of four droplet size settings ranging from Very Fine to Very Coarse, while tier two expands the options to aircraft type and setup, swath width and displacement, droplet size data, and meteorology. Where appropriate, these parameters were set to those used in the field. AgDISP modeling included selecting aerial as the method, with the aircraft set as an Air Tractor 802A. Spray pressure, release height, number and spacing of nozzles, and total swath width were set to those used in the field study (Table 4) and the droplet size data were directly imported as the incremental volume diameter distribution from wind tunnel testing. The study setup used two different orifice size across the boom, at a constant ratio of two 0.078 orifice sized nozzles to each 0.125 orifice sized nozzle. However, AgDISP does not allow for mixed nozzle types within a single simulation run. To account for the different droplet sized sprays and flow rates from each orifice size, two model iterations were run, one for each orifice size. The deposition results for each run were then combined weighting each set of results by the percentage of the total application flow rate corresponding to each orifice size, with 41.9 and 58.1% of the total flowrate contributed by the 0.078 and 0.125 orifice nozzles, respectively. As previously stated, AgDISP defaults to a half swath offset; however, in this study the aerial spray passes were offset a full swath upwind, which is not a standard option in the model. However, the user can specify a swath displacement value, which the model adds to the specified swath offset, meaning for the aerial modeling, the swath offset was set to zero and the swath displacement set to 21.9 m.

### Statistical analysis

All field spray drift data and soybean response data were analyzed by fitting four parameter log-logistic functions (Eq. 1) using the “drc” package in R4.0.3^65^:

1 $$f\left(x\right)=c+ \frac{d-c}{1+\mathrm{exp}(b\left(\mathrm{log}\left(x\right)-\mathrm{log}\left(e\right)\right))}$$

In which *f(x)* is the specific response variable, *b* is the slope at the inflection point, *c* is the lower limit, *d* is the upper limit, *x* is the downwind distance from the spray application, and *e* is the inflection point. A variance ratio (*F*-test) was conducted to determine whether individual or pooled application-type models best fit the data (*P* ≤ 0.05)^66^. For all soybean response variables, as data were percentages bound between 0 and 100%, the lower limit (c) and upper limit (*d*) were fixed in the models at 0 and 100, respectively. Models were subsequently used to predict the estimated downwind distance in which the respective response variable was reduced by 25, 50, and 90% (PD_25_, PD_50_, and PD_90_, respectively).

Droplet size data are presented as D_v10_, D_v50_, and D_v90_, which are the droplet diameters at which 10, 50, and 90%, respectively, of the total spray volume is comprised of smaller diameter droplets. Additionally, the percent fines are reported as the percent volume of the spray contained in droplets of 100 μm or less. No means comparisons were made as it is known that the different nozzle types and operational conditions would result in sprays of differing droplet size.

### Experimental research on plants statement

The authors declare that the cultivation of plants throughout the presented research complies with all relevant institutional, national, and international guidelines and legislation. Further, the seed specimens used in this study are publicly accessible seed materials, and the authors were given explicit written permission to use them for research purposes.

## Data Availability

The datasets generated and analyzed during the current study are available from the corresponding author on reasonable request.
